# Influence of *GRK5* gene polymorphisms on ritodrine efficacy and adverse drug events in preterm labor treatment

**DOI:** 10.1038/s41598-020-58348-1

**Published:** 2020-01-28

**Authors:** Jee Eun Chung, Jeong Yee, Han Sung Hwang, Jin Young Park, Kyung Eun Lee, Young Ju Kim, Hye Sun Gwak

**Affiliations:** 1 College of Pharmacy and Institute of Pharmaceutical Science and Technology, Hanyang University, Ansan, South Korea; 2 College of Pharmacy and Graduate School of Pharmaceutical Sciences, Ewha Womans University, Seoul, South Korea; 3 Department of Obstetrics and Gynecology, Konkuk University Medical Center, Konkuk University School of Medicine, Seoul, South Korea; 4 College of Pharmacy, Chungbuk National University, Seoul, South Korea; 5 Department of Obstetrics and Gynecology, Ewha Womans University School of Medicine, Seoul, South Korea

**Keywords:** Predictive markers, Genetics research

## Abstract

The present prospective follow-up study aimed to evaluate the effects of *GRK5* polymorphisms on ritodrine efficacy and adverse drug events (ADEs) in pregnant women undergoing preterm labor. A total of 162 women undergoing preterm labor were included in the study. Seven single nucleotide polymorphisms (SNPs) in the *GRK5* gene (rs915120, rs2230345, rs2230349, rs7923896, rs1020672, rs4752308, and rs4752292) were assessed. Homozygous variant carriers of rs4752292 and rs1020672 had 0.6 times the hazard of delivery compared to wild-type allele carriers (95% confidence interval [CI], 0.41~0.99 and 0.38~0.99, respectively). In addition, homozygous variant carriers of rs4752292 and rs1020672 had 2.4-fold more (95% CI, 1.10~4.98) and 2.3-fold more (95% CI, 1.04~5.06) ADEs compared to those with the wild-type homozygotes, respectively. Among demographic variables, gestational age and modified Bishop score were significant factors associated with time to delivery, while body weight and maximum ritodrine infusion rate were significant factors associated with ADEs. *In silico* analysis showed that both rs4752292 and rs1020672 had the potential to affect mRNA splicing by alteration of splicing motifs. The present study shows that ritodrine efficacy and ADEs are associated with *GRK5* gene polymorphisms in pregnant women undergoing preterm labor.

## Introduction

Preterm birth, defined as birth before the usual 37 weeks of pregnancy, is one of the main causes of perinatal mortality and morbidities that persist into childhood^1^. As of now, upon diagnosis of preterm labor, tocolytic agents have been frequently utilized for the prolongation of pregnancy. Although the pathogenesis of preterm labor and functional mechanism of tocolytics are yet to be fully understood, it has been reported that many tocolytics act via G-protein coupled receptor (GPCR) signal-mediated pathways involved in myometrium relaxation and contraction^2^. Control of uterine contractility involves numerous neurotransmitters and hormones, which act through membranous receptors that activate intracellular signaling pathways. Beta adrenergic receptors activated by catecholamines exhibit a key function in the mediation of uterine relaxation, as can be observed by the role of beta agonists such as ritodrine in preterm labor treatment^3,4^.

Beta agonists bind and activate beta 2 adrenergic receptors located on the outer membrane of myometrial cells, increasing cAMP levels and thus resulting in a decrease of intracellular Ca^2+^ levels and uterine contraction^5^. Our previous study showed that *ADRB2* polymorphisms affected beta agonist efficacy in pregnant women undergoing preterm labor^6^. Genetic polymorphisms in the beta adrenergic receptor are assumed to alter the binding affinity of beta agonists and consequently, the corresponding intracellular signaling pathways.

In addition, one of the important mechanisms mediating events following GPCR activation is receptor desensitization. Receptor desensitization is an adaptive process where cell responsiveness to extensive agonist stimulation is decreased, whereas reaction to other agonists or activators is unaffected. Desensitization commences with GPCR uncoupling, mediated by two different types of serine/threonine kinases; G protein-coupled receptor kinases (GRKs) and second messenger-dependent kinases, including cAMP-dependent protein kinase A (PKA) or protein kinase C (PKC)^7^. GRKs trigger homologous desensitization via the selective phosphorylation of agonist-bound GPCRs, while PKA and PKC initiate heterologous desensitization^8^. In the process of desensitization after beta agonist action, beta receptor phosphorylation is initiated by GRKs and beta arrestin binds to the complex. Sterically blocking G protein activation leads to beta adrenergic receptor endocytosis and desensitization. Therefore, GRKs are important regulators of GPCR function and mediate receptor desensitization, internalization, and signaling^8^.

GPCR kinases are a class of protein kinases encoded by *GRK* genes, which are composed of the genes *GRK1* to *GRK7*. The seven *GRK* subtypes can be classified into three groups in a sequence homology-dependent fashion^9^. Members of the *GRK1* family (*GRK1* and *GRK7)* show retina-specific expression, while those of the *GRK2* family (*GRK2* and *GRK3*) exhibit a broader distribution. Among the *GRK4* family members, *GRK4* is localized to the testis, while *GRK5* and *GRK6* display ubiquitous expression^8,9^.

*In vivo* studies on *GRK* gene polymorphisms have been carried out in the context of several disease areas including cardiovascular disease, neuropsychiatric disease, and diabetes^10–14^. Uterine smooth muscle is regulated by the beta adrenergic pathway, and among the *GRKs, GRK5* is highly expressed in the human myometrium^15,16^. However, there has been limited study of the effects of *GRK5* polymorphisms on the myometrium.

Therefore, this study aimed to investigate the relationship between *GRK5* polymorphisms and ritrodine efficacy and adverse drug events (ADEs) in pregnant women undergoing preterm labor.

## Methods

### Participants and data collection

The study was conducted at Ewha Womans University Mokdong Hospital in a prospective fashion, from January 2010 to December 2014^17^. Criteria for eligibility of participation were as follows: age ≥18 years, preterm labor with intact membranes, gestational age of 20–36 weeks, and uterine contractions at a frequency of three per 10 min with cervical changes. Patients with the following high-risk conditions upon admission were excluded; pre-eclampsia, placental abruption, fetal distress, fetal/placental/amniotic abnormalities, placenta previa, severe spontaneous premature rupture of membranes, clinical chorioamnionitis or major vaginal bleeding. Patients treated with ritodrine to prevent uterine contractions during McDonald operation or treated with tocolytics other than ritodrine were excluded. All procedures performed in studies involving human participants were in accordance with the ethical standards of the institutional and/or national research committee and with the 1964 Helsinki declaration and its later amendments or comparable ethical standards. The study was approved by the Ethics Committee and Institutional Review Board of Ewha Womans University Medical Center (IRB number: 217-1-26). Informed consent was obtained from each participant before the study.

The outcomes of therapy and clinical information were recorded and collected from electronic medical records. The primary endpoints were time to delivery and proportion of women who remained undelivered. In addition, ritodrine-induced ADEs were investigated as a secondary endpoint. ADEs were defined as cases of drug cessation or dose reduction due to presence of tachycardia, palpitations, dyspnea, shortness of breath, or pulmonary edema. Patient information included maternal age, body weight, height, gestational age, comorbidity, modified Bishop score, smoking status, time of initiation and termination of ritodrine therapy, ritodrine dose, and type of adverse event. All data generated or analysed during this study are included in this published article (and its Supplementary Information files).

### Drug administration

Ritodrine (Lavopa^®^; JW Pharmaceutical, Seoul, Korea) was administered via intravenous infusion at an initial rate of 0.05 mg/min which was increased by 0.05 mg/min every 10 min until the desirable uterine response was obtained. Intravenous treatment was discontinued during uterine quiescence. Patients who achieved uterine quiescence received maintenance therapy with an infusion of 0.05 mg/min for 12–48 h.

### Genotyping

Blood samples were collected for genotyping during admission. Genomic DNA was extracted from ethylenediaminetetraacetic acid (EDTA)-blood samples using the QIAamp DNA Blood Mini Kit (QIAGEN GmbH, Hilden, Germany) following the manufacturer’s recommendations. *GRK5* SNPs were selected based on previous studies and genetic information from UCSC genome browser^10,13^. In addition, genetic information about the *GRK5* gene was incorporated into the software Haploreg ver 4.2 for the selection of *GRK5* SNPs with a minor allele frequency (MAF) of ≥20% in the Japanese and Han Chinese populations. The linkage disequilibrium blocks were constructed following the D’-method^18^. One SNP in 5’ UTR (rs7923896), two missense SNPs (rs2230345 and rs2230349), and four intronic SNPs (rs915120, rs1020672, rs4752308 and rs4752292) were selected. A total of seven SNPs were genotyped using SNaPShot Multiplex kits.

### Statistical analysis

The interval from start of ritodrine therapy to delivery was analyzed using the Kaplan-Meier survival data analysis method (log-rank test). Cox’s proportional-hazards model was used for exploratory multivariate analysis. Categorical variables were analyzed using the chi-squared test or the Fisher’s exact test. A multivariable logistic regression model was used to identify independent predictors using factors with a p-value < 0.1 in univariate analysis. The fit of the prediction model was assessed using the Hosmer-Lemeshow goodness-of-fit test. Discrimination of the model was further assessed via area under receiver operating curve (AUROC) analysis, which assessed the ability of risk factors to predict ritodrine-induced ADEs. On the basis of Rozenberg *et al*.’s study^19^ and the assumption that overall observed MAFs of chosen SNPs were 30%, the post-hoc power analysis was conducted with PROC Power of SAS 9.4 (SAS Institute, Cary, NC, USA). All statistical tests were conducted with a two-tailed alpha of 0.05. The data were analyzed using the Statistical Package for Social Sciences Version 20.0 for Windows (SPSS, Chicago, IL, USA).

### In silico analyses

To predict the possible effects of given variants on splicing, different computational tools were used. Netgene2^20^ and Splice Site Prediction by Neural Network (NNSPLICE)^21^ were used for splice site predictions. Alternations of the splicing factor binding site pattern caused by the given mutation were evaluated using Exonic Splicing Enhancer (ESE) finder 3.0^22^ and Human Splicing Finder (HSF) 3.1^23^. We used the default threshold values, and a score for a given sequence was considered to be potentially significant, if it was above the threshold values.

## Results

A total of 216 women treated with ritodrine were enrolled. Sixteen who had already experienced severe symptoms, 10 who were scheduled for the McDonald operation, eight who had underlying cardiovascular diseases, and 20 without blood samples were excluded. A total of 162 patients were ultimately included in our study, and the post hoc power analysis showed that our study had 77% power to detect a clinically meaningful decrease of 35% in time to delivery. The mean maternal age was 31.0 (± 3.7) years, and the mean gestational age at initiation of drug therapy was 29.6 (± 3.9) weeks. The maximum infusion rate was 0.10 ± 0.06 mg/min. Multiple gestation pregnancies were observed in 12.1% of the study population. There was no current smoker. Among demographic variables, gestational age (p < 0.001), modified and Bishop score (p = 0.012) were significantly associated with median time to delivery (Table 1).

**Table 1:** Effects of demographic variables and *GRK* polymorphisms on time to delivery.

Characteristics		No of patients (n = 162)	Time to delivery Median (95% CI)	P value
Demographic factors				
Age	<30 years	57	1168.6 (772.8–1564.4)	0.383
	≥30 years	105	1082.1 (775.5–1388.7)	
Weight	<60 kg	62	995.8 (426.5–1565.1)	0.865
	≥60 kg	100	1190.7 (901–1480.4)	
Height	<160 cm	58	978.8 (787–1170.6)	0.376
	≥160 cm	104	1190.7 (938–1443.4)	
BSA	<1.65	77	978.6 (744.5–1212.7)	0.529
	≥1.65	85	1230.6 (998.4–1462.8)	
Gestational age	<28 weeks	56	2004.5 (1679.5–2329.5)	<0.001
	≥28 weeks	106	828.6 (649.8–1007.4)	
Modified Bishop score^†^	<2	84	1298.9 (1143.3–1454.5)	0.012
	≥2	41	613.9 (0–1309.2)	
Multiple pregnancy	1	123	1072.6 (817–1328.2)	0.544
	≥2	17	884.9 (85.9–1683.9)	
*GRK5* gene polymorphisms				
rs915120 (T > C)	TT, TC	103	1082.1 (705.5–1458.7)	0.316
	CC	55	1190.7 (878.9–1502.5)	
rs2230345 (A > T)	AA, AT	151	1168.6 (928.5–1408.7)	NA
	TT	0	NA	
rs2230349 (G > A)	GG, GA	155	1072.6 (808.1–1337.1)	0.767
	AA	4	1082.1 (0–2419.6)	
rs7923896 (C > T)	CC	65	859.2 (620.4–1098)	0.354
	CT, TT	93	1238.2 (975.9–1500.5)	
rs4752292 (T > G)	TT,TG	72	905.3 (608.2–1202.4)	0.041
	GG	88	1265.0 (1028.7–1501.3)	
rs1020672 (C > T)	CC, CT	66	828.6 (497.5–1159.7)	0.012
	TT	90	1300.6 (1152.7–1448.5)	
rs4752308 (T > C)	TT	49	991.5 (633.4–1349.6)	0.292
	TC, CC	110	1168.6 (811.4–1525.8)	

^†^Modified Bishop score is the sum of dilatation score and effacement score. Dilatation score 0, <l cm; 1, 1–3 cm; 2, 3–5 cm; 3, ≥5 cm. Effacement score 0, 0–40%; 1, 40–60%; 2, 60–80%; 3, ≥80%. NA, not available.

**Figure 1:**
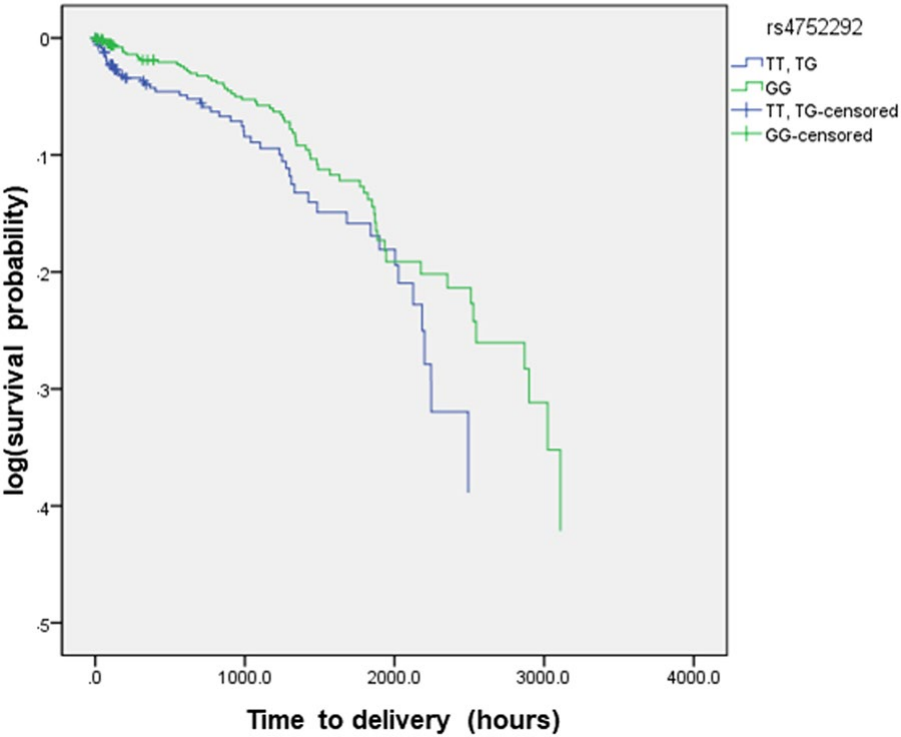
Kaplan–Meier survival analysis of the effect of *GRK5 r*s4752292, comparing the TT and TG group with the GG group (p = 0.041).

The effects of seven SNPs in the *GRK5 g*ene on ritodrine efficacy were evaluated. All of the allele frequencies were consistent with the Hardy-Weinberg equilibrium. Two polymorphisms (rs1020672 and rs4752292) were in linkage disequilibrium (LD) in our study population (r^2^ = 0.8). In univariate analysis, rs4752292 (T > G) and rs1020672 (C > T) were significantly associated with time to delivery. Homozygous variant carriers of rs4752292 and rs1020672 had a longer median time to delivery than those with the other genotypes (Table 1, Fig. 1).

For multivariate analysis, two models were constructed using factors with P < 0.1 from the results of univariate analysis in addition to age, because rs1020672 and rs4752292 were in LD. The SNP rs4752292 was used in Model I and the SNP rs1020672 in Model II. The models revealed that homozygous variant carriers of rs4752292 and rs1020672 had 0.6 times the hazard of delivery compared to wild-type allele carriers (95% confidence interval [CI], 0.41~0.99 and 0.38~0.99, respectively). In addition, women at a gestational age ≥28 weeks and modified Bishop score ≥2 had 6.8~8.9 and 1.8~1.9 times the hazard of delivery, respectively, compared to the other women in the study (Table 2).

**Table 2:** Multivariate Cox regression models of time to delivery.

Factor	Model I		Model II	
	Hazard Ratio (95% CI)	*p* Value	Hazard Ratio (95% CI)	*p* Value
Age (≥30 years)	0.98 (0.62–1.55)	0.922	0.98 (0.62–1.56)	0.929
Gestational age at start of drug therapy (≥28 weeks)	6.81 (3.48–13.33)	<0.001	8.92 (4.27–18.62)	<0.001
Modified Bishop score (≥2)	1.92 (1.23–3.01)	0.004	1.77 (1.11–2.82)	0.016
*GRK5* rs4752292 Homozygous variant (GG)	0.64 (0.41–0.99)	0.047		
*GRK5* rs1020672 Homozygous variant (TT)			0.62 (0.38–0.99)	0.045

Adjusted for factors with *p*-value < 0.1 from results of univariate analysis in addition to age.

In terms of the proportion of women who remained undelivered at 24 h and/or at seven days after start of ritodrine therapy, a statistically significant difference was present in rs4752292 carriers at seven days and in rs1020672 carriers at 24 h and at seven days. Carriers of the GG genotype of rs4752292 had an approximately 20.6% greater proportion who remained undelivered at seven days compared to wild-type allele carriers (p = 0.002). On the other hand, carriers of the homozygous variant TT of rs1020672 were 100% undelivered at 24 h and 14.5% greater proportion who remained undelivered at seven days compared to wild-type allele carriers (Table 3).

**Table 3:** Effects of *GRK5* gene polymorphisms on proportion of patients who remained undelivered.

*GRK5* Gene polymorphism	Grouped genotypes	24 h		Seven days	
		Proportion (%)	P value	Proportion (%)	P value
rs915120	TT, TC	96.9	0.799	83.0	0.826
	CC	96.2		81.4	
rs2230345	AA, AT	96.5	NA	82.4	NA
rs2230349	GG, GA	96.6	0.708	81.9	0.349
	AA	100.0		100.0	
rs7923896	CC	96.7	1.000	80.4	0.646
	CT, TT	96.7		83.5	
rs4752292	TT, TG	94.1	0.107	71.2	0.002
	GG	98.8		91.8	
rs1020672	CC, CT	93.5	0.017	75.0	0.030
	TT	100.0		89.5	
rs4752308	TT	93.6	0.156	76.7	0.231
	TC, CC	98.1		85.2	

In univariate analysis of ritodrine-induced ADEs, weight (p = 0.009), height (p = 0.002), and body surface area (BSA) (p = 0.003) were significant factors associated with incidence of ADEs (Table 4). Women who experienced ADEs had lower weight and height than those without ADEs. Further, the maximum ritodrine infusion rate was a marginally significant factor for ADEs. Among the SNPs studied, rs4752292 was the only significantly associated variant and marginal significance was observed in case of rs1020672.

**Table 4:** Effects of demographic variables and *GRK* polymorphisms on ritodrine-induced adverse events.

		No adverse event group (n = 119)	Adverse event group (n = 43)	P value
Demographic factors				
Age (years)		30.99 ± 3.48	30.95 ± 4.21	0.954
Weight (kg)		63.32 ± 8.24	59.47 ± 8.22	0.009
Height (cm)		161.51 ± 4.4	159.07 ± 4.61	0.002
BMI (kg/m^2^)		24.29 ± 3.12	23.47 ± 2.77	0.130
BSA		1.68 ± 0.12	1.62 ± 0.13	0.003
Gestational age (weeks)		29.82 ± 3.89	29.02 ± 3.87	0.249
Modified bishop score		1.29 ± 1.44	0.97 ± 1.34	0.269
Maximum infusion rate (mL/min)		5.63 ± 3.15	6.77 ± 3.84	0.057
*GRK5* polymorphism				
rs915120	TT, TC	79 (68.1)	24 (57.1)	0.201
	CC	37 (31.9)	18 (42.9)	
rs2230345	AA, AT	112 (100)	39 (100)	NA
rs2230349	GG, GA	114 (96.6)	41 (100)	0.573^a^
	AA	4 (3.4)	0(0)	
rs7923896	CC	49 (41.9)	16 (39)	0.749
	CT, TT	68 (58.1)	25 (61)	
rs4752292	TT, TG	59 (50)	13 (31)	0.033
	GG	59 (50)	29 (69)	
rs1020672	CC, CT	54 (46.6)	12 (30)	0.068
	TT	62 (53.4)	28 (70)	
rs4752308	TT	37 (31.4)	12 (29.3)	0.803
	TC, CC	81 (68.6)	29 (70.7)	

^a^Relative risk.

**Figure 2:**
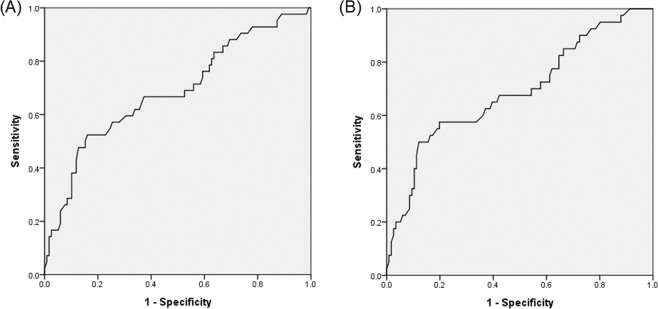
Area under receiver operating characteristic curve representing ritodrine-induced adverse drug events. (**A**) Model I included weight, maximum infusion rate, and rs4752292, and the AUROC value was 0.703 (95% CI, 0.605~0.801). (**B**) Model II included weight, maximum infusion rate, and rs1020672, and the AUROC value was 0.689 (95% CI, 0.589~0.789).

Multivariate logistic regression was performed using factors with P < 0.1 from the results of univariate analysis in addition to age. As height, weight and BSA were related to each other, multivariate logistic regression was performed only with weight, which could represent the remaining variables, to consider multicollinearity in the analysis. Similar to the multivariate analysis of time to delivery, two models were constructed, because rs1020672 and rs4752292 were in LD. The rs4752292 polymorphism was used in Model I and rs1020672 in Model II. As shown in Table 5, after adjusting for covariates, carriers of the homozygous variant rs4752292 and rs1020672 had 2.35-fold (95% CI, 1.10~4.98) and 2.29-fold (95% CI, 1.04~5.06) higher incidence of ADEs than those with other genotypes. The risk of ADEs increased 1.1-fold per 1 mL/min increase in maximum infusion rate whereas it decreased 0.96-fold per 1 kg increase in body weight. The Hosmer-Lemeshow test revealed good fits of the models (Model I, χ^2^ = 8.61, P = 0.376; Model II, χ^2^ = 8.38, P = 0.398). The AUROC values of Model I and Model II were 0.703 (95% CI, 0.605~0.801) and 0.689 (95% CI, 0.589~0.789), respectively (Fig. 2).

**Table 5:** Logistic regression analysis of risk factors associated with adverse reaction to ritodrine.

Factor		Model I		Model II	
		Odds Ratio (95% CI)	*P* value	Odds Ratio (95% CI)	*P* value
Weight		0.96 (0.95–0.98)	<0.001	0.96 (0.95–0.98)	<0.001
Maximum infusion rate		1.11 (1.01–1.22)	0.036	1.13 (1.02–1.24)	0.018
*GRK5* rs4752292	TT, TG	1	0.027		
	GG	2.35 (1.10–4.98)			
*GRK5* rs1020672	CC, CT			1	0.039
	TT			2.29 (1.04–5.06)	

Adjusted for factors with *p-*value < 0.1 from results of univariate analysis in addition to age.

Analysis of two SNPs (rs4752292 and rs1020672) with Netgene2 and NNSPLICE showed a significant association with ritodrine efficacy and ADEs, and did not show the presence of an altered splicing donor or acceptor. However, the results generated by HSF 3.1 indicated that rs4752292 disrupted two RESCUE-ESE hexamers (AGACAT and CATCAG) and two exon-identity elements (EIEs) hexamers (ACATCA and TCAGAT) (Supplementary Table 1). It also led to the formation of SRp40, SF2/ASF (IgM-BRCA1), and SF2/ASF enhancing motifs (AGACAGC, CAGCAGA, and CAGCAGA); the scores were 83.71 (threshold: 78.08), 77.00 (threshold: 70.51), and 78.10 (threshold: 72.98), respectively. The analysis by ESEfinder 3.0, with a different scoring Falgorithm from that of HSF 3.1, also showed that this mutation increased the score of SRp40 binding from 1.588 to 3.611 (threshold: 2.67), SF2/ASF (IgM-BRCA1) binding from 1.012 to 2.670 (threshold; 1.867), and SF2/ASF binding from 0.762 to 2.826 (threshold: 1.956), creating new enhancer motifs. According to the results of HSF 3.1, rs1020672 was predicted to break two Sironi’s Motif 3 and create four intron-identity elements (IIEs) hexamers (CCTCTT, CTCTTT. CTTTTC, and TTTTCA).

## Discussion

The present study is the first to suggest that *GRK5* variations are correlated with beta agonist efficacy and risk of ADEs in women undergoing preterm labor, the key finding being that homozygous variant carriers of the *GRK5* polymorphisms rs4752292 and rs1020672 showed 0.6 times the hazard of delivery compared to wild-type carriers. The proportion of women who remained undelivered at seven days after initiation of ritodrine therapy was significantly higher in homozygous variant carriers of rs4752292 and rs1020672 than in wild-type allele carriers. All women with the homozygous variant TT of rs1020672 remained undelivered at 24 h. Furthermore, those with variant-type homozygous genotypes in the two SNPs above showing higher ritodrine efficacy experienced 2.3~2.4-fold increased ADEs than others.

In the rat and human myometrium, activation of the beta adrenergic receptor/adenyl cyclase pathway via catecholamines significantly induces myorelaxation during pregnancy. At the end of pregnancy, the beta adrenergic receptor/adenyl cyclase pathway is desensitized, contributing to contraction initiation at parturition^2,24,25^. Receptor desensitization has been reported to rely on alterations in beta adrenergic receptor coupling rather than in receptor expression^24^. An animal study showed that the abrupt decrease in adenyl cyclase response to beta agonist stimulation occurring just prior to parturition was caused by concomitant beta adrenergic receptor-Gs protein uncoupling, and increased myometrial GRK activity^26^. These results supported the notion that GRK may function as a physiological regulator of myometrial beta adrenergic receptor activity. Within the GRK family, GRK5 is abundantly expressed in the myometrium. A study with GRK-specific monoclonal antibodies showed that, among several subtypes of GRK, GRK5 and/or GRK6 mediated β-adrenergic desensitization^27^. Because of the high basal phosphorylation and autophosphorylation activity of GRK5, it was thought to desensitize the β-adrenergic receptors even without hormonal activation, which importantly involved in maintaining uterine quiescence^28^. Thus, polymorphisms in *GRK5* may have a crucial role in beta agonist response.

In human studies, polymorphisms in *GRK* have been studied extensively in terms of cardiovascular diseases. *GRK5* polymorphisms have shown a significant association with atrial fibrillations and heart failure. The SNP rs4752292, a significant factor identified in our study, has been suggested to be correlated with the risk of postoperative atrial fibrillation^29^. Kertai *et al*. confirmed that this *GRK5* variation had a strong association with postoperative atrial fibrillation despite using beta blockers in patients undergoing coronary artery bypass graft surgery^30^. Although rs1020672 has not been previously studied, our results show that this SNP is in LD with rs4752292 and that it has a significant association with ritodrine efficacy.

The SNPs of rs4752292 and rs1020672 are located in intronic regions, which are not thought to be involved in the production of proteins. However, intronic regions may potentially influence mRNA splicing, gene expression, or protein activity; thus, analyzing these regions may be useful^31,32^. To evaluate the spicing effects of these two SNPs, we employed various computational tools and found that both SNPs were predicted to affect the splicing of the *GRK5* gene. Therefore, rs4752292 and rs1020672 may be candidate SNPs that affect ritodrine efficacy and ADEs.

A functional SNP (rs2230345, Gln41Leu) in the *GRK5* gene was reported to blunt the effects of catecholamines via enhanced beta receptor desensitization *in vitro* and in clinical settings^10,12,33^. A study showed that the polymorphism exhibited improved survival in patients with heart failure taking beta blockers^33^. Another study on airway smooth muscle, in which GRK5 is highly expressed, revealed that the variant allele of rs2230345 induced increased agonist-promoted desensitization of adenylyl cyclase compared with the wild allele. Hence, it was thought that the Leu41 variant could increase kinase function, which results in loss-of-function in the beta 2 adrenergic signaling pathway, thereby decreasing bronchodilatory effects by beta agonist therapy^34^. Unfortunately, the variant allele of rs2230345 was rarely found in our study population, although it is known to be prevalent in African Americans.

As the secondary endpoint, we analyzed the association between *GRK5* polymorphisms and ADE induced by ritodrine. Similar to their effects on efficacy, the SNPs rs4752292 and rs1020672 were found to be significant factors after adjusting for variables. The result showed that women with homozygous variants of either SNP had 2.3~2.4-fold higher risk of ritodrine-induced ADEs. This suggests that the polymorphisms rs4752292 and rs1020672 may alter desensitization of GPCRs and affect both ritodrine efficacy and ADEs.

Among demographic factors, gestational age and modified Bishop score were significant factors for time to delivery, while weight and maximum ritodrine infusion rate influenced on ritodrine-induced ADEs. However, we could not find the impacts of ex-smoking, due to the lack of information on smoking history. Although the significance of these demographic factors may be expected, more specific studies to understand the mechanisms leading to differences in responses to beta agonists are required.

Based on our results, two SNPs of GRK5 (rs4752292 and rs1020672) showed associations with both efficacy and ADRs of ritodrine. They could be good candidates for discriminating the patients who sufficiently responded to ritodrine or who are particularly vulnerable to ADRs. Additionally, our results can be extended to a study of pharmacogenomic association between GRK5 and β-agonist bronchodilators for treatment of asthma or chronic obstructive pulmonary disease.

## Conclusions

The present pharmacogenomics study suggests that genetic polymorphisms in *GRK5* affect individual susceptibility to beta 2 adrenergic receptor-targeted therapy in women undergoing preterm labor. Because ethnical diversity of study population was not acquired, further studies including other populations are recommended.

## Supplementary information

Supplementary Info.

Dataset.
