# Supplementary material for: Influence of GRK5 gene polymorphisms on ritodrine efficacy and adverse drug events in preterm labor treatment
Source: Sci Rep. 2020 Jan 28;10:1351. doi: 10.1038/s41598-020-58348-1 (PMC6987149; doi:10.1038/s41598-020-58348-1)
Supplement: Supplementary file 1 — Supplementary Info. [file 41598_2020_58348_MOESM1_ESM.docx]

**Influence of *GRK5* gene polymorphisms on ritodrine efficacy and adverse drug events in preterm labor treatment**

Jee Eun Chung^1*^, Jeong Yee^2*^, Han Sung Hwang^3^, Jin Young Park^2^, Kyung Eun Lee^4^, Young Ju Kim^5^, Hye Sun Gwak^2^

^1^College of Pharmacy and Institute of Pharmaceutical Science and Technology, Hanyang University

^2^College of Pharmacy and Graduate School of Pharmaceutical Sciences, Ewha Womans University

^2^Department of Obstetrics and Gynecology, Konkuk University Medical Center, Konkuk University School of Medicine

^4^College of Pharmacy, Chungbuk National University

^5^Department of Obstetrics and Gynecology, Ewha Womans University School of Medicine

^*^The authors equally contributed to this work.

Corresponding author: Hye Sun Gwak

College of Pharmacy and Division of Life & Pharmaceutical Sciences, Ewha Womans University, 52 Ewhayeodae-gil, Seodaemun-gu, Seoul 03760 Republic of Korea

Tel: +82-2-3277-4376; Fax: +82-2-3277-3051; E-Mail: hsgwak@ewha.ac.kr

Co-corresponding author: Young Ju Kim

Department of Obstetrics and Gynecology, Ewha Womans University School of Medicine 1071 Anyangcheon-ro, Yangcheon-gu, Seoul 07985 Republic of Korea

Tel: +82-2-2650-5209; Fax: +82-2-2653-8891; E-Mail: kkyj@ewha.ac.kr

**Supplementary Materials**

Supplementary Table 1. Potential splicing regulatory sequences in wild-type and mutant-type of rs4752292 and rs1020672 by Human Splicing Finder (HSF) 3.1.

Supplementary Data 1 (separate excel file)**.** Datasets for analysis of patients.

Supplementary Table 1. Potential splicing regulatory sequences in wild-type and mutant-type of rs4752292 and rs1020672 by Human Splicing Finder (HSF) 3.1.

| Type of signal | Matrice | Linked motif or SR (threshold) | Wild-type sequence (Score) | Mutant-type Sequence (Score) | | Variation (%) |
| --- | --- | --- | --- | --- | --- | --- |
| **rs4752292 (NM_005308.2:c.148+14030T>G)** | | |  |  |  | |
| Splice acceptor site | HSF Matrices | TTGCCCAGACATCA | 54.26 | ttgcccagacagCA (83.21) | | New site |
|  |  | CCGAGACATCAGAT | 80.51 | cccagacagcagAT (80.55) | | +0.05 |
| Branch Point | HSF Matrices | ACATCAG | 67.3 | 52.21 | | Site broken |
|  |  | ATCAGAT | 68.47 | 67.26 | | -1.77 |
| Enhancer Motif | ESE finder | SF2/ASF (IgM-BRCA1) (70.51) | CAGACAT (79.38) | CAGACAG (77.85) | | -1.94 |
|  |  | SF2/ASF (IgM-BRCA1) (70.51) | CAGACAT (79.38) | CAGACAG (79.32) | | -0.08 |
|  |  | SF2/ASF (72.98) | CAGACAT (81.54) | CAGACAG (77.85) | | -4.53 |
|  |  | SF2/ASF (72.98) | CAGACAT (81.54) | CAGACAG (79.32) | | -2.71 |
|  |  | SRp40 (78.08) | - | AGACAGC (83.71) | | New site |
|  |  | SF2/ASF (IgM-BRCA1) (70.51) | - | CAGCAGA (77.00) | | New site |
|  |  | SF2/ASF (72.98) | - | CAGCAGA (78.10) | | New site |
|  | RESCUE-ESE hexamers | - | AGACAT | - | | Site broken |
|  |  | - | CATCAG | - | | Site broken |
|  |  | - | ATCAGA | AGCAGA | | - |
|  | PESE octamers | - | AGACATCA (28.37) | - | | Site broken |
|  | EIEs hexamers | - | AGACAT | AGACAG | | - |
|  |  | - | GACATC | GACAGC | | - |
|  |  | - | ACATCA | - | | Site broken |
|  |  | - | ATCAGA | AGCAGA | | - |
|  |  | - | TCAGAT | - | | Site broken |
|  | HSF Matrices | 9G8 (59.245) | GACATC (59.46) | GACAGC (62.89) | | +5.76 |
|  |  | 9G8 (59.245) | - | GCAGAT (69.73) | | New site |
| Silencer Motif | Sironi motifs | Motif 1 (CTAGAGGT; 60) | CCAGACAT (67.01) | - | | Site broken |
| **rs1020672 (NM_005308.2:c.53-1294C>T)** | | |  |  |  | |
| Splice acceptor site | HSF Matrices | CCCTCCTTTCAGCT | - | ccctcttttcagCT | | -0.46 |
|  | MaxEntScan | 3’ Motif (3) | CTTGTTTTCCCTCCTTTCAGCTT (11.56) | cttgttttccctcttttcagCTT (11.12) | | -3.81 |
| Branch Point | HSF Matrices | CTTTCAG | 70.07 | 68.54 | | -2.18 |
| Silencer Motif | Sironi motifs | Motif 3 (TCTCCCAA; 60) | CCTCCTTT (65.96) | - | | Site broken |
|  |  | Motif 3 (TCTCCCAA; 60) | CCTTTCAG (61.36) | - | | Site broken |
|  | IIEs hexamers | - | - | CCTCTT | | New Site |
|  |  | - | - | CTCTTT | | New Site |
|  |  | - | TCCTTT | TCTTTT | | - |
|  |  | - | - | CTTTTC | | New Site |
|  |  | - | - | TTTTCA | | New Site |

EIE, exon-identity element; IIE, intron-identity element; PESE, predicted exonic splicing enhancer.
